# Facile Synthesis and Characterization of Chitosan Functionalized Silver Nanoparticles for Antibacterial and Anti-Lung Cancer Applications

**DOI:** 10.3390/polym15122700

**Published:** 2023-06-16

**Authors:** Devaraj Bharathi, Jaya Ganesh Thiruvengadam Nandagopal, Jintae Lee, Rajamani Ranjithkumar

**Affiliations:** 1 School of Chemical Engineering, Yeungnam University, Gyeongsan 38541, Gyeongbuk, Republic of Korea; jtlee@ynu.ac.kr; 2 Department of Biotechnology, Bharathiar University, Coimbatore 641046, Tamil Nadu, India; van.thamizhan@gmail.com; 3 Viyen Biotech LLP, Coimbatore 641031, Tamil Nadu, India; 4 Department of Biotechnology, Sri Ramakrishna College of Arts & Science, Nava India, Coimbatore 641006, Tamil Nadu, India

**Keywords:** Ag NPs, chitosan, TEM, antibacterial activity, anticancer agent

## Abstract

In the treatment of bacterial contamination, the problem of multi-drug resistance is becoming an increasingly pressing concern. Nanotechnology advancements enable the preparation of metal nanoparticles that can be assembled into complex systems to control bacterial and tumor cell growth. The current work investigates the green production of chitosan functionalized silver nanoparticles (CS/Ag NPs) using *Sida acuta* and their inhibition efficacy against bacterial pathogens and lung cancer cells (A549). Initially, a brown color formation confirmed the synthesis, and the chemical nature of the synthesized NPs were examined by UV-vis spectroscopy, Fourier transform infrared spectroscopy (FTIR), scanning electron microscopy (SEM) coupled with energy dispersive spectroscopy (EDS), and transmission electron microscopy (TEM). FTIR demonstrated the occurrence of CS and *S. acuta* functional groups in the synthesized CS/Ag NPs. The electron microscopy study exhibited CS/Ag NPs with a spherical morphology and size ranges of 6–45 nm, while XRD analysis demonstrated the crystallinity of Ag NPs. Further, the bacterial inhibition property of CS/Ag NPs was examined against *K. pneumoniae* and *S. aureus*, which showed clear inhibition zones at different concentrations. In addition, the antibacterial properties were further confirmed by a fluorescent AO/EtBr staining technique. Furthermore, prepared CS/Ag NPs exhibited a potential anti-cancer character against a human lung cancer cell line (A549). In conclusion, our findings revealed that the produced CS/Ag NPs could be used as an excellent inhibitory material in industrial and clinical sectors.

## 1. Introduction

Infectious diseases are the leading cause of premature death on a global scale [1,2]. At the same time, cancer is the second leading cause of early death globally [3]. Many infectious illnesses have developed resistance to commercial antibiotics and alternative remedies [4]. The growth of drug resistance has become a major concern for humans and also for pharma industries [5]. Thus, the search for new and efficient inhibitory drugs against microbial infections has intensified. Over the past few decades, nanoparticles (NPs) have demonstrated an ability to eradicate several drug-resistant infections and diseases [6]. Among the many metal and metal oxide NPs, Ag NPs have attracted interest due to their distinctive characteristics [7]. From ancient times, Ag and Ag-based compounds have been recognized for their bactericidal characteristics [8]. Ag NPs have been utilized to prevent microbiological contamination in the textile, cosmetic, and food sectors using current science and technology [9]. There are many physical, chemical, and biological methods available for synthesizing Ag NPs [10,11,12]. During the preparation of Ag NPs by physico-chemical processes, the utilization of harmful reagents, the need for high temperatures, and the emission of toxic byproducts have all had a negative impact on biological applications [13,14].

Hence, an appropriate and green alternative to the chemical and physical route of synthesis is biological methods. Living organisms, such as bacteria, plants, fungi, algae, and even animals, play a vital role in the synthesis of NPs because of some constituent biomolecules, including amino acids, vitamins, phytocompounds, and other secondary metabolites, that can be extracted from them and help in the in-situ reduction of Ag+ ions to form Ag NPs. The primary advantage of biosynthesis is the process’s safety and the purity of resulting NPs. We can be certain that the product synthesized would be free of contamination because only benign substances are utilized to aid the production of Ag NPs using this method. This has a low influence on human health, especially as these NPs are employed for a variety of medicinal applications. Aside from that, biological approaches provide a high production of NPs that are well-defined in shape and homogeneous in size: sometimes even more so than physiochemical procedures. The utilization of plants and their parts as possible factories for the production of Ag NPs is a relatively new and promising field of research [15,16,17]. Potential applications have been identified for the preparation of Ag NPs when utilizing plants such as *Platycladus orientalis* [18], *Mangifera indica* [19], *Jatropha integerrima* [20], *Lippia citriodora* [21], and *Manilkara zapot* [22]. Recently, Takci et al. [23] demonstrated the potential bactericidal character of Ag NPs when synthesized using *Salvia officinalis* against *P. aeruginosa*, *E. coli*, *S. typhimurium,* and *S. aureus*. In a recent investigation, Sharma et al. [24] revealed that phyto-synthesized Ag NPs utilizing a *Callistemon lanceolatus* leaf extract exhibited a dosage-dependent bactericidal effect against *S. aureus*, *B. subtilis*, *M. luteus*, *E. coli,* and *K. pneumoniae*. Pavan et al. [25] reported that Ag NPs developed using *Dictyota ciliolate* exhibited a potential anticancer activity against A549 lung cancer cells.

In this study, an aqueous *S. acuta* Burm.f. leaf extract was used in the production of CS/Ag NPs. *S. acuta* is a member of the Malvaceae family and is frequently known as wireweed. *S. acuta* has a long history of use as a medicinal plant, and it has been effective against a variety of illnesses. *S. acuta* possesses antibacterial, antioxidant, anti-inflammatory, antimalarial, insecticidal, and anti-plasmodial activities, according to pharmacological studies [26,27]. In recent years, the green synthesis of organic-inorganic NPs has been a developing subject in nanoscience and nanotechnology due to its sustainable uses in the biopharmaceutical, food, and clinical sectors [28,29]. Organic-inorganic nano-complexes, such as polymer-coated metal nanoparticles have attracted considerable attention due to their low toxicity, eco-friendliness, simple production procedures, and good surface binding characteristics [30]. Among the different combinations of organic-metal NPs, CS functionalized Ag NPs represent a novel class of nano-complexes with enhanced characteristics, properties, and uses [31]. Chitosan (CS) is a natural polymer that is generated from the deacetylation of chitin and possesses unique features, including biodegradability, biocompatibility, and antibacterial capabilities [32,33]. A study by Wongpreecha et al. [34] reported that one-pot synthesized Ag NPs-CS exhibited effective bactericidal properties against *E. coli* and *S. aureus*. Another study by Gobinath et al. [35] documented that biocompatible CS-decorated Ag NPs showed potential anticancer activity against A549 cells.

**Figure 1:**
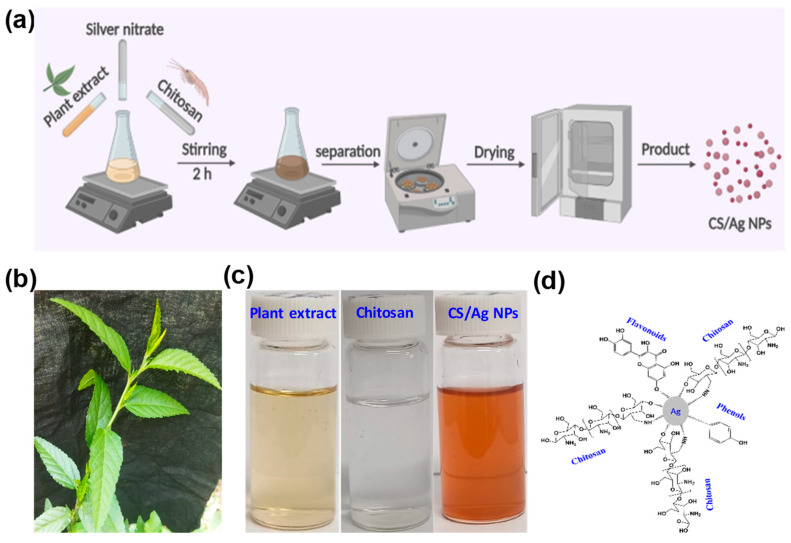
(**a**) Schematic depiction of the synthesis approach to develop CS/Ag NPs using *S. acuta*, (**b**) *S. acuta* image, (**c**) Colorization images of *S. acuta*, CS and CS/Ag NPs and (**d**) Chain network formation of CS/Ag NPs from CS and *S. acuta* leaf extract.

The green production of Ag NPs has been broadly investigated; however, those for the synthesis of CS-loaded Ag NPs with increased properties and potential applications are still challenging. Thus, the current study was designed to synthesize CS/Ag NPs using *S. acuta* by an eco-friendly route (Figure 1). Our investigation followed a facile protocol to generate CS/Ag NPs using the leaf extract of *S. acuta* without extra reagents, solvents, precipitating agents, and templates. Further, CS/Ag NPs were characterized by various physical and chemical techniques, and assessed for their antibacterial activity against *K. pneumonia* and *S. aureus* by well diffusion and dual staining fluorescence method. Moreover, the anticancer activity of CS/Ag NPs was investigated against a human lung cancer cell line (A549) by the MTT assay, and its apoptosis induction ability was also analyzed.

## 2. Experimental Sections

### 2.1. Chemicals and Cultures

Chitosan (deacetylation degree ≥ 75%, molecular weight; 190–375 kDa), silver nitrate, 3-[4,5-96 dimethylthiazole-2-yl]-2, 5-diphenyltetrazolium bromide (MTT), and Muller-Hinton agar were procured from HiMedia, Mumbai, India. The fresh leaves of *S. acuta* were obtained from Bharathiar University and authenticated by the Botanical Survey of India (Reference number: BSI/SRC/5/23/2020/651). The bacterial strains (*K. pneumoniae* and *S. aureus*) were acquired from a clinical lab in Coimbatore.

### 2.2. Extraction of S. acuta Leaf Samples

The harvested leaves were initially cleaned, dried for four days, and ground into a fine powder. A Soxhlet extractor was utilized to obtain an aqueous extract by combining 10 g of fine powder with 100 mL of distilled water. Then, the extracted substance was kept at 4 °C until further usage.

### 2.3. Synthesis of CS/Ag NPs

The synthesis of CS/Ag NPs was carried out by a green chemistry method, as previously reported by Nandana et al. [30], with some modifications. About 10 mL of the *S. acuta* extract and 10 mL of CS solution (0.1 g of CS in 10 mL of 1% acetic acid) were mixed with 80 mL of a 1 mM AgNO_3_ solution, and then the solution was agitated for 2 h at room temperature under a dark state. After this, the resulting colloidal solution was configured at 15,000 rpm for 10 min before being separated by washing with distilled water to remove unbound *S. acuta* compounds and CS in the synthesized CS/Ag NPs and dried at 80 °C.

### 2.4. Physico-Chemical Characterization

#### 2.4.1. Optical Studies

UV-vis spectroscopy (JASCO-V-670) was subjected to observe the reaction mixture as it underwent the formation of CS/Ag NPs. The visible spectra of NPs were observed from 300 to 800 nm.

#### 2.4.2. FTIR

The IR analysis was used to identify the molecules of chitosan and *S. acuta* that were accountable for the formation of NPs. Readings from the FTIR (FTIR-00585, PerkinElmer) spectrum were taken with a resolution of 4 cm^−1^ across the range of 400–4000 cm^−1^.

#### 2.4.3. SEM-EDS

The SEM was used to characterize the NPs’ morphology (JEOL-Model JSM 6390 coupled with EDS). In order to prepare a SEM sample on a grid, a pinch of NPs was positioned on the grid then the surfaces of the sample were sputter-coated with carbon tape and then imaged. This experiment was carried out with a voltage of 10 kV applied to the accelerator.

#### 2.4.4. TEM

The structure of synthesized NPs was observed using TEM. A very small pinch of NPs was placed on a grid, and NPs images were observed at 120 kV (JEOL-Japan).

### 2.5. Disc Diffusion Antibacterial Activity Assay

The bactericidal activity of CS/Ag NPs was assessed against *K. pneumoniae* and *S. aureus* using a disc diffusion assay [36]. Briefly, the chosen bacterial strains (10^5^ CFU/mL) were spreaded on the MHA plates. Then, the empty discs were filled with various concentrations of CS/Ag NPs (10, 20 and 30 µg/mL) and allowed to dry for a few minutes. Then, coated discs were positioned on the dish and kept at 37 °C for 24 h to observe the activity.

### 2.6. AO/EtBr Dual Staining and Growth Curve Assay

The changes in the *K. pneumonia* and *S. aureus* cell membrane upon action with NPs were examined using an AO/EtBr staining technique, as described by Kumar et al. [37]. Briefly, 1 mL of overnight-grown pathogens were incubated with 20 µg/mL of NPs for 24 h at 37 °C. The cells without NPs were kept as a control. Then, the cells were cleaned with a buffer solution and further stained with 1 µL of AO/EtBr, and images of fluorochemical staining were attained using a Nikon Eclipse Ni-E Microscope with a Nikon DS-Ri2 digital camera. The digitized images were processed using Nikon’s proprietary software NIS-Elements BR.

### 2.7. Growth Curve Analysis

The growth curve analysis of synthesized CS/Ag NPs was tested against the chosen pathogens (*K. pneumoniae* and *S. aureus*) [38]. Briefly, overnight cultures of *K. pneumoniae* and *S. aureus* were incubated with 20 µg/mL of CS/Ag NPs, and the O.D. was monitored at every 4 h interval up to 24 h.

### 2.8. Anticancer MTT Assay against A549 Cells

Human lung cancer cells were obtained from the National Centre for Cell Science (NCCS), Pune, India. Initially, the monolayer cell culture was trypsinized, and the cell amount was attuned to 1 × 10^4^ cells/mL with 10% FBS medium. About 0.2 mL of the diluted cell suspension was added to each well of the 96-micro titer plate. When a partial monolayer formed after 24 h, the supernatant was flicked off, and the monolayer was washed once with a buffer. Different concentrations of CS/Ag NPs (20, 40, 60, 80, and 100 μg/mL) being diluted in media were added and incubated for 24 h to determine the effect of CS/Ag NPs on cell viability. After that 10 µL of MTT (5 mg/mL) was added and further incubated for 4 h, then removed, and a further 200 µL of DMSO was added and thoroughly mixed to dissolve the dark blue crystals. Then, the absorbance was measured at 570 nm, and the percentage of viability was calculated using Formula (1).

Cell viability (%) = O.D. of test/O.D. of control × 100(1)

### 2.9. Apoptosis Induction Studies

Apoptosis causing ability of synthesized CS/Ag NPs was studied using a fluorescence microscopic dual staining method as previously described by Bharathi et al. [39]. About 300 μL of lung cancer cells were treated with the IC_50_ concentration of CS/Ag NPs in a 16-well plate and incubated for 24 h. After that, the treated and untreated cells were washed with PBS and stained with 10 μL of an AO/EtBr mixture before the morphology changes were imaged using a fluorescence microscope (Nikon Eclipse Ni-E Microscope with a Nikon DS-Ri2 digital camera).

### 2.10. Statistical Analysis

The experimental investigation, including testing for antibacterial and anticancer activities, was performed in triplicates, and the findings were reported as the mean ± standard deviation. The statistical significance of the differences were determined using a *p*-value of 0.05.

## 3. Results and Discussion

### 3.1. Formation of CS/Ag NPs

The green production of CS/Ag NPs was carried out using *S. acuta* leaves extract (Figure 1b). Previously, Uysal et al. [27] reported that *S. acuta* contained various phytochemical properties such as hydroxybenzoic, hydroxycinnamic and acylquinic acids, hydroxycinnamoyl tartarates, flavonoids, cinnamic acid amides, alkaloids, and amino acids. The presence of these phytocompounds in *S. acuta* and CS might have acted as reducing and capping agents for the synthesis of CS/Ag NPs [39]. The development of NPs was established by the color change from yellow to brown (Figure 1c). The development of this brown color was well known to be emerged from SPR vibrations of nano-CS/Ag [40]. Similarly, various phytocompounds of *Myristica fragrans* [41], *Muntingia calabura* [42], and *Rumex nervosus* [43] mediated and synthesized Ag NPs and exhibited brown color formation. The mechanism of this chain network formation by CS and phyto-compounds with Ag is shown in Figure 1d. The active phyto-compounds presented in *S. acuta* and CS acted as ligation agents. The existence of long pair of e^−^, free -NH_2_, and O-H groups in CS and phytocompounds of *S. acuta* might have ligated with Ag^2+^ and developed an Ag–CS–ellagate complex. Then, this network system naturally led to a nucleation process that went into reverse micellization, which further rooted the reduction of Ag ions (Ag^+^) to nano Ag [30,44,45].

### 3.2. Characterization Studies

#### 3.2.1. Optical Studies

**Figure 2:**
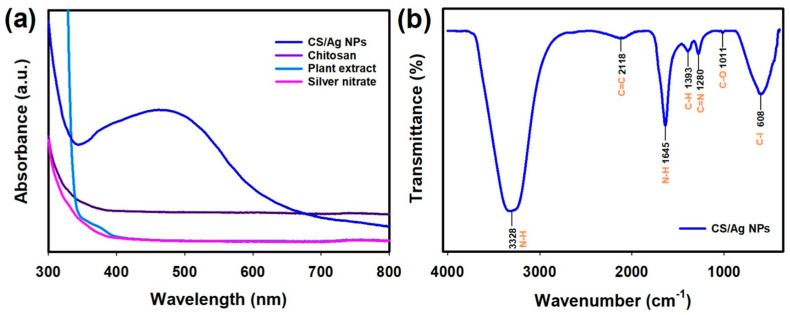
(**a**) UV−Visible spectroscopy analysis of silver nitrate, plant extract, CS and synthesized CS/Ag NPs: The CS/Ag NPs were examined in cuvettes with a route length of one centimeter across the range of 300 to 800 nm, and (**b**) FTIR fingerprint of synthesized CS/Ag NPs: The resolution for the IR study was set at 4 cm^−1^, and the analysis was performed across the range of 4000–400 cm^−1^.

The UV-vis spectroscopy investigation of CS/Ag NPs exhibited an absorbance peak at 464 nm and thus supported the synthesis of CS/Ag NPs (Figure 2a). According to reports, the UV absorbance peak around 400–480 nm was a distinct property of nano-Ag [46]. Similarly, CS-*Aegle marmelos* entrapped Ag NPs exhibited UV-vis absorbance at 420 nm [47]. The UV-visible spectrum of CS-decorated Ag NPs was prepared using *Piper betle* exhibited an absorption peak at 430 nm [35]. Recently, Ag NPs were synthesized using CS, and seaweed showed the UV-vis absorbance peak to be around 425 nm [48].

#### 3.2.2. FTIR-Functional Groups Investigation

The attraction of active groups and the development of end product CS/Ag NPs were studied using FTIR. The IR analysis of CS/Ag NPs is depicted in Figure 2b. The FTIR of NPs showed a typical primary N-H band at 3328 cm^−1^, C=C vibration at 2118 cm^−1^, N-H band at 1645 cm^−1^, C-H bending at 1393 cm^−1^, C-N band at 1280 cm^−1^, C-O stretching at 1011 cm^−1^ and a C-I band at 608 cm^−1^. The obtained N-H and C-O functional groups could be derived from -NH_2_ groups and acetylated parts of CS [49]. Other groups, namely C=C, C-H, C-N, and C-I, may be derived from the phyto-extract of *S. acuta*. Moreover, certain band vibrations around 600–400 cm^−1^ could be accredited to the presence of metal (Ag) in the synthesized CS/Ag NPs. The presence of these bio-active derivatives might have acted as a reductant for the formation of final CS/Ag NPs [36].

#### 3.2.3. Microscopic and EDS Analysis

**Figure 3:**
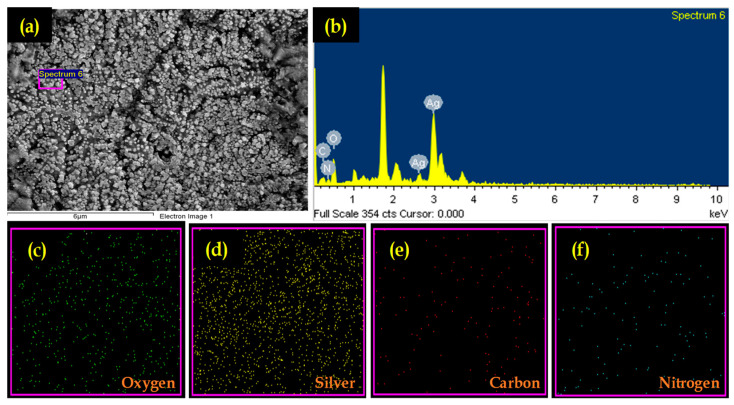
Electron microscopic analysis of synthesized CS/Ag NPs using *S. acuta* leaf extract: (**a**) SEM image, (**b**) EDS analysis and (**c**–**f**) mapping of O, Ag, C and N, respectively. The X-ray emission peaks of Ag, O, C and N are labelled.

Figure 3a shows the FE-SEM of CS/Ag NPs. FE-SEM images showed that the NPs had a spherical morphology. Ag NPs were synthesized using various plant extracts, which showed a spherical-shaped morphology [50,51]. Further, EDS was performed to analyze the elemental composition of CS/Ag NPs (Figure 3b). The elemental spectra of CS/Ag NPs exhibited a peak of silver (Ag), which supported the formation of Ag NPs. The other peaks of C, N, and O were detected due to the emission of X-rays from the -NH_2_ and de-acetylated groups of CS. The elemental percentage in the synthesized CS/Ag NPs is given in Table 1. Similar to our study, Nandhana et al. reported on the presence of Ag, N, C, and O in the green synthesized CS/Ag nanocomposite using rutin. Further, elemental mapping analysis confirmed the random distribution of observed elements in the synthesized NPs (Figure 3e,f).

#### 3.2.4. TEM Analysis

**Figure 4:**
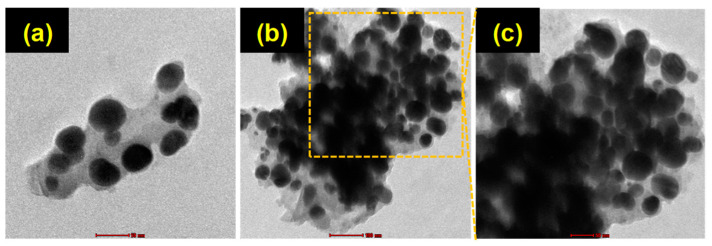
(**a**−**c**) TEM images of synthesized CS/Ag NPs at different magnifications. An experiment using a TEM was carried out with an accelerator voltage of 100 kV.

Further, the morphology and size of produced NPs were analyzed using TEM. The TEM images of synthesized CS/Ag NPs are shown in Figure 4a–c. TEM showed the particle sizes varied from 6 to 45 nm, and it also showed the synthesized NPs to have a spherical morphology with Ag crystallinity. Similar to our study, gallic acid-chitosan-modified Ag NPs exhibited a spherical-shaped morphology [52].

### 3.3. Disc Diffusion Bactericidal Assay

**Figure 5:**
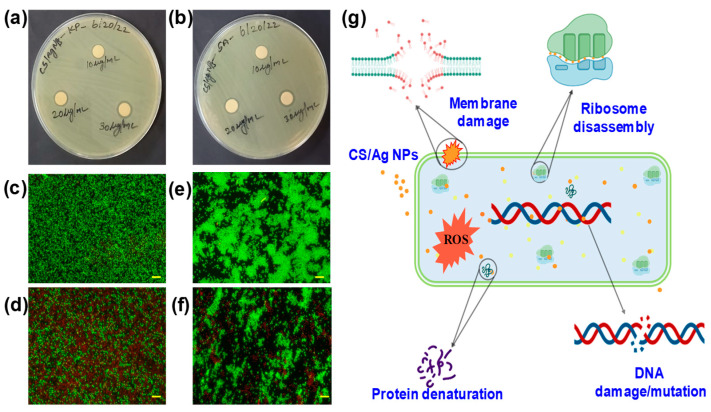
Antibacterial activity assays: disc diffusion antibacterial plates of (**a**) *K. pneumonia,* (**b**) *S. aureus* treated with CS/Ag NPs at different concentrations (10, 20 and 30 µg/mL), fluorescence microscopic images of the control and CS/Ag NPs treated (**c**,**d**) *K. pneumonia* and (**e**,**f**) *S. aureus*, respectively, and (**g**) proposed antibacterial mechanism of CS functionalized Ag NPs. In order to evaluate the samples, sterile discs with a diameter of 5 mm were utilized, and the ZOI was determined in millimeters of diameter. The AO/EtBr dual fluorescent dye was used for fluorescent staining, and the results showed that the green, fluorescent color represented alive cells while the red fluorescent color depicted dead cells.

The antibacterial properties of CS/Ag NPs were investigated against *K. pneumoniae* and *S. aureus*. The synthesized NPs inhibited potential and concentration-dependent inhibitory activities against the tested bacterial pathogens. Figure 5a,b depicts a well diffusion plate experiment with various concentrations (10, 20, and 30 µg/mL) of CS/Ag NPs. Against all the pathogens examined, clear zones of bacterial suppression appeared around the CS/Ag NPs embedded discs. At an increasing concentration of NPs, the bactericidal ZOI increased. The ZOI for *K. pneumoniae* was found to be 9 ± 0.5 mm for 10 µg/mL, 11 ± 0.8 mm for 20 µg/mL and 13 ± 0.2 for 30 µg/mL of CS/Ag NPs, whereas *S. aureus* exhibited 10 ± 0.2 mm for 10 µg/mL, 11 ± 0.6 mm for 20 µg/mL, and 13 ± 1 mm for 30 µg/mL. Synthesized NPs exhibited equal activity for both tested pathogens. The results of NPs’ action could change depending on the bacteria’s cell wall and membrane structure [36]. Moreover, nano-scale CS possessed a significant bactericidal and broad-range antibacterial action against bacterial infections [53]. Similar to our study, CS-Ag NPs coated linen fabrics showed their potential antibacterial activity against *E. coli* and *S. aureus* [54].

### 3.4. Dual Fluorescent Staining

**Figure 6:**
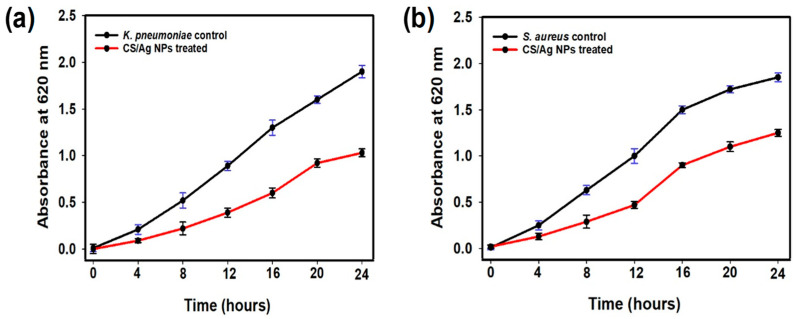
Growth curve analysis of CS/Ag NPs: (**a**) *K. pneumoniae* and (**b**) *S. aureus*. Data denote the mean (±) standard errors of three independent assays.

Furthermore, the antibacterial efficacy of produced CS/Ag NPs against *K. pneumoniae* and *S. aureus* was validated by utilizing a fluorescent-based live/dead cell test. The fluorescent microscopic pictures of the control and NPs treated cells are shown in Figure 5c–f. The untreated cells fluoresced green due to the existence of live cells, whereas treated cells fluoresced red, confirming their death. Fluorescent AO is a green dye that stains both live and dead bacteria, whereas EtBr is a red dye that exclusively stains dead bacteria. The EtBr penetrates dead cells via the cell membrane and reduces the green fluorescent color of AO [55]. Accordingly, the green color denotes living bacterial cells, whereas the red hue signifies dead bacterial cells. The obtained results revealed that produced NPs had a bacteriostatic impact against the tested bacterial pathogens. Further, the growth curve analysis revealed that CS/Ag NPs were able to prevent the growth of *K. pneumoniae* and *S. aureus* and also showed delayed growth (Figure 6).

A potential mechanism for the bacterial suppression of CS/Ag NPs is shown in Figure 5g. One of the two fundamental processes that may be responsible for the inhibition action of Ag-based NPs is the breakdown of cell walls and membrane destruction (1). The attraction and interaction between NPs and bacteria begin with Ag adhesion to the plasma membrane, which causes membrane structural alterations, resulting in membrane depolarization, disruption of permeability, and disruption of cell wall integrity. As a consequence of depolarization, the internal material of bacterial cells escapes into the surrounding environment, leading to the death of the cells. The second approach involves the creation of reactive oxygen species (ROS), which can include superoxide, hydroxyl radicals, and hydrogen peroxides, among other things. These limit the development of bacteria by binding to the genetic elements and proteins that are found on their cells [56,57,58].

### 3.5. Anticancer Studies

#### 3.5.1. MTT Assay

The anticancer property of CS/Ag NPs was assessed against human lung cancer cells using a MTT assay. The obtained findings exhibited a significant decrease in the growth of A549 cells (97.2%) when the concentration of CS/Ag NPs increased. CS/Ag NPs induced a 50% growth defeat property at a concentration of 34.5 ± 0.5 µg/mL. Similarly, Murugesan et al. [47] reported that the synthesized Ag NPs using *Gloriosa superba* exhibited potential anticancer activity against A549 cells. Similar findings were reported by Priya et al. [59], who observed the dose-dependent anticancer activity of biogenic CS-Ag NPs against hepatocellular carcinoma cells. The biogenic synthesized Ag NPs entrapped with CS showed anticancer activity against HeLa cells [47]. It was reported that the anticancer properties of Ag NPs were dependent on the morphology, size, and reducing agents of the NPs.

#### 3.5.2. AO/EtBr Fluorescent Assay

**Figure 7:**
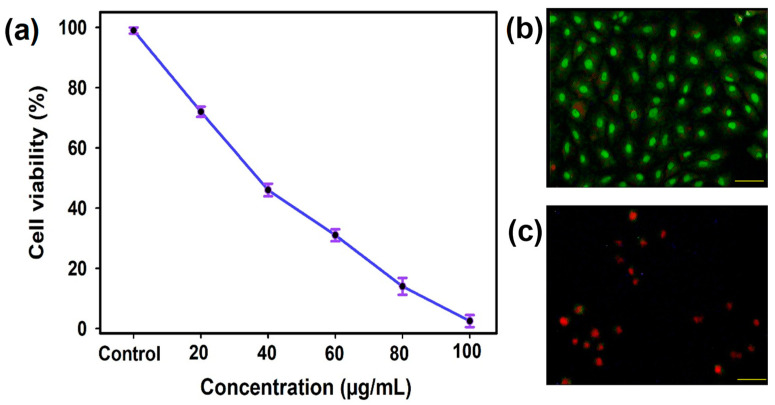
Anticancer activity of CS/Ag NPs against human lung cancer cell line (A549): (**a**) MTT assay, and fluorescent images of (**b**) control cells, and (**c**) treated cells. The data shown here are the means and standard errors obtained from three separate tests.

Nearly all unicellular creatures undergo apoptosis, which is a type of planned cell death. A helpful strategy for the therapy of cancer is the induction of apoptosis [60]. Using a fluorescence microscope, the apoptosis cells were separated from one another by their orange or red-colored bodies. Fluorescence microscope images revealed that the prepared CS/Ag NPs could induce apoptosis in treated A549 cells. The control cells displayed a green color, and the treated cells showed a red color, thus supporting the induction property of CS/Ag NPs (Figure 7a,b). The appearance of a red hue indicated the presence of apoptotic bodies and displayed cell shrinkage as well as membrane blebbing. In most cases, the morphological and biochemical alterations in such cell shrinkage, membrane blebbing, membrane unity, nuclear fragmentation, and cytoplasmic condensation contributed to cell death by initiating the apoptotic pathway. This process was responsible for the death of cells. [61,62]. The apoptosis induction property of NPs depended on the permeability of Ag ions into the cancer cells, which stimulated cell damage, and DNA fragmentation and, thus spontaneously led to apoptosis [63].

## 4. Conclusions

Silver is one of the most often utilized metals that is accessible and has a diverse variety of applications. In biomedical applications, the nanoform of Ag, also known as Ag NPs, has provided a novel structure for particles. The synthesis of Ag NPs using chemical methods is both expensive and harmful; thus, efforts have been made to utilize biological sources such as plants, bacteria, and algae in order to cut down on both costs and toxicity. In the present investigation, *S. acuta* performed outstandingly both as a reducing and capping agent. The production of CS/Ag NPs using the *S. acuta* aqueous leaf extract has been presented as an eco-friendly and simple green technique. To our knowledge, this is the first report on the synthesis of CS/Ag NPs using *S. acuta* without any additional chemicals and reagents. The size of the prepared CS/Ag NPs ranged from 6 to 45 nm and had a spherical form. The occurrence of CS in prepared CS/Ag NPs was validated by EDS and FTIR analyses. Significant antibacterial activity was exhibited by the synthesized CS-Ag NPs against *K. pneumonia* and *S. aureus* pathogens. In addition, the MTT and fluorescent-based assay confirmed its anti-cancer capabilities with the apoptosis induction property of synthesized CS/Ag NPs against the human lung cancer cell line (A549). The potential antibacterial and anticancer properties of CS/Ag NPs could be due to the synergetic properties of both CS and Ag NPs. Thus, we believe that the CS/Ag NPs synthesized using *S. acuta* have the potential to be employed as NPs in clinical sectors to reduce the growth of bacterial and also cancer cells.

## Figures and Tables

**Table 1:** The percentage of elements present in the CS/Ag NPs.

Elements	Weight(s)
Ag	53
C	02
N	14
O	31

## Data Availability

Not applicable.
